# Institutional Questions

**Published:** 1899-10-07

**Authors:** 


### INSTITUTIONAL QUESTIONS.

### Convalescent Day-Rooms.

A correspondent writes: We are about to make some structural alterations in our village hospital, and should be glad of advice on one or two points. 1. As to the provision of a day-room for convalescent patients. Do you consider this addition to accommodation sufficiently necessary to justify some extra expense? 2. The floors of the wards are in a bad state. They are of ordinary unpolished deal. Would you advise entire re-flooring, and if so, of what material?

1. The provision of a day-room for convalescents is most desirable, and your committee will certainly do well to arrange for its inclusion in their scheme of improvements. It should, if anyway possible, face south or south-east, so as to catch as much sunshine as may be, and made a bright and cheerful place. Woodwork should be painted white or some light shade, and the introduction of yellow tones into the decoration generally, helps to give a sunny effect. We have seen this done recently in a convalescent home, and it is surprising how greatly the details of fittings aid in securing the general result aimed at. An appeal to residents in the neighbourhood should secure a supply of pictures for the walls, and books and papers, &c. 2. With regard to flooring opinions widely differ as to the best material, and a good deal depends upon special circumstances in each case. It is of little use endeavouring to improve old floors, and in the long run the best plan will probaby be to re-lay these entirely. Oak is likely to prove the most satisfactory material, but everything depends upon the way the boards are laid. They should be "side" nailed, so that no nail-holes appear on the surface, and wax-polished.

### Homes for the Dying.

"F. M." asks: Kindly give me the names of "Homes for the Dying" in London.

There are several, and you had better apply direct for particulars of terms of admission, as you do not mention the circumstances of the patient on whose behalf you write. Friedenheim Home of Peace, Sunnyside, Upper Avenue Road, Swiss Cottage, N.W.; National Free Home for the Dying, 82, The Chase, Clapham Common, S.W. (under the care of the Sisters of St. Margaret, East Grinstead); St. Luke's House, 50, Osnaburgh Street, N.W. (in connection with the West London Mission). There is also a Home for the Dying at Wimbledon (offices, 281, Strand, W.C.), but of this we have no particulars. Other homes for incurable and chronic cases you will find in "Hospitals and Charities" (Scientific Press), p. 940.

### Fever Hospital.

A "Londoner" writes: Is the London Fever Hospital, Islington, one of the Metropolitan Asylums Board institutions, and what are the terms of admission for ordinary patients?

The London Fever Hospital has no connection with the Metropolitan Asylums Board. It is supported by voluntary contributions and by patients' payments. Scarlet fever cases are treated at all times so far as accommodation allows, and diphtheria, measles, and typhoid when the space available permits. Ward patients pay a fee of £3 3s., a sum equal to about a fourth of their cost, the balance of expense falling upon the hospital funds. Private rooms may be had at £3 3s. a week.

